# Identification and characterization of potent CD-19 scFv and CD-69 expression in CAR J Cells

**DOI:** 10.12669/pjms.42.(ICON26).15687

**Published:** 2026-04

**Authors:** Shakir Hussain, Muhammad Zulqurnain Imran, Talha Israr, Javeria Aijaz, Yasir Raza

**Affiliations:** 1 Shakir Hussain Deputy Manager, Molecular Pathology, Indus Hospital & Health Network, Karachi, Pakistan; 2 Dr. Muhammad Zulqurnain Imran Consultant, Department of Hematology, Indus Hospital & Health Network, Karachi, Pakistan; 3 Talha Israr Assistant Manager, Department of Hematology, Indus Hospital & Health Network, Karachi, Pakistan; 4 Dr. Javeria Aijaz Senior Consultant, Molecular Pathology, Indus Hospital & Health Network, Karachi, Pakistan; 5 Dr. Yasir Raza Assistant Professor, Department of Microbiology, University of Karachi, Karachi, Pakistan

**Keywords:** Chimeric antigen receptor (CAR), CD19, T-cell activation, B-cell acute lymphoblastic leukemia (B-ALL)

## Abstract

**Background and Objective:**

CD19 is the primary target for generating chimeric antigen receptor (CAR) T cells against B-cell leukemia. This study aimed to test a screening protocol for evaluating the efficacy of the CAR constructs using patient-derived B-ALL cells and CAR–expressing Jurkat (CAR-J) cells.

**Methodology:**

This study was conducted in the Molecular Research Department of Indus Hospital, Karachi, Pakistan, from January 2024 to August 2025. CAR-containing backbone plasmids (#135991, #135992, and #135993) obtained from Addgene were used to produce second-generation VSV-G pseudotyped lentiviruses. Jurkat cells were infected with these lentiviruses to create CAR-J cells. CAR-J cells were cocultured with patient-derived B-ALL CD-19-positive cells. Flow cytometry was used to evaluate CD69-APC MFI expression following co-culture (a marker of Jurkat cell activation), using APC-labelled antibodies. Increased CD69 expression in comparison with controls (uninfected Jurkat cells, acute myeloid leukemia) was taken as an indicator of the efficacy of CAR binding with CD19.

**Results:**

Only two of the three anti-CD19 CAR-J constructs induced CD69 upregulation upon co-culture with patient-derived B-ALL cells (CAR-J 135992 and CAR-J 135993) in comparison with AML controls (p = 0.0044 and p = 0.0195, respectively). When compared with uninfected Jurkat cocultured with B-ALL, all three CAR-J constructs showed significant CD69 upregulation: 135991 (p = 0.0004), 135992 (p = 0.0063), and 135993 (p = 0.0187).

**Conclusion:**

Two anti-CD19 CAR-J cell constructs (135992 and 135993) demonstrated a detectable increase in CD69 upregulation compared with AML controls, while one construct (135991) showed no activation. Once further validated, the method may have potential for screening of other CAR molecules.

## INTRODUCTION

B-cell lymphoproliferative disorders have antigens on their surface, which are used as a target for the Chimeric Antigen Receptor (CAR) immunotherapy for patients, i.e., CD22 and CD19, etc. Immunotherapies utilize CD19 and CD22 as primary targets present on B-cell acute lymphoblastic leukemia blasts.^1^ T-Cells have specific surface receptor proteins, which enable these cells to target specific antigens. These (TCR) or chimeric antigen receptors are receptor proteins that have been modified (engineered) to give T cells the new ability to target specific antigens.^2^

CAR T cells are T cells that have been genetically modified (engineered) to produce an artificial T cell receptor for use in immunotherapy.^3^ These CAR T cells detect surface antigens without relying on MHC. When they bind to tumor-specific antigens, they multiply and destroy the tumor cells.^4^ CARs consist of an antigen-recognition domain, a single-chain variable fragment (scFv ligand) derived from a monoclonal antibody, along with a costimulatory domain and a CD3 signaling domain from the T-cell receptor (TCR). In second-generation CARs, this signaling region also incorporates a costimulatory domain.^5^ Each CD19-directed CAR T-cell therapy is unique in its design and manufacturing process. These differences arise from variations in the CAR backbone, including the choice of signaling and costimulatory domains, which influence the activation strength and persistence of CAR T cells. Additionally, gene transduction methods, such as lentiviral, retroviral, or non-viral systems, impact the stability and expression level of the CAR construct. Culture conditions during T-cell expansion, including cytokine composition and activation protocols, further affect the phenotype, functionality, and durability of the final CAR T-cell product. Collectively, these factors contribute to differences in clinical efficacy, safety profile, and durability of response among CAR T-cell therapies, even when they target the same antigen, such as CD19.^6^,^7^

Jurkat cells, an immortalized human T-cell leukemia line, are widely used as a model system for studying T-cell activation and signaling. While they do not fully mimic the cytokine profile or cytolytic function of primary T cells, they are capable of producing interleukin-2 (IL-2) and upregulating early activation markers such as CD69 upon stimulation. Due to the ease of detecting CD69 expression via fluorescent antibody staining and flow cytometry, this marker has been frequently used in in vitro studies to evaluate CAR-mediated T-cell activation, making Jurkat cells a practical tool for preliminary CAR construct screening. The CAR-J screening platform using CD69 as an activation marker in Jurkat cells has been previously validated.^8^-^12^ All of these studies have, however, used cell lines for these validations.

In this study, we evaluated a method for determining the efficacy of chimeric antigen receptors against their targets using patient-derived mononuclear cells instead of established cell lines. The primary aim of this adaptation is to determine if the screening method already in use with cell lines is effective if these are replaced with actual patient-derived cells. If so, this adaptation may have relevance in the future to test for effective CAR against individual patients, in addition to the use of screening for novel CAR.

## METHODOLOGY

This study was conducted in the Molecular Research Department of Indus Hospital & Health Network, Karachi, Pakistan, from January 2024 to August 2025. Patients were included through convenient sampling method. Three de-identified samples from confirmed B-ALL patients. were included. Each sample was analyzed in triplicate to ensure reproducibility. All relevant data were obtained from hospital records.

### Ethical approval:

The study was approved by the Institutional Review Board (IHHN_IRB_2021_12_019; dated November 28, 2024) of Indus Hospital & Health Network (IHHN). Written informed consent was obtained from all participants prior to sample collection. All patient specimens were fully de-identified before analysis, and no personal identifiers were linked to the experimental data

### Inclusion criteria:

Patients diagnosed with B-cell acute lymphoblastic leukemia (B-ALL) confirmed by flow cytometry and positive for CD19 antigen expression were included in the study.

### Exclusion criteria:

Samples with insufficient cell viability after isolation or those with dim CD19 expression were excluded.

### Cell culture:

**Fig.1:**
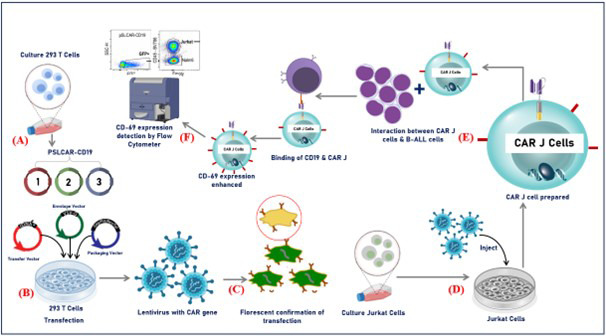
Experiment design including, (A) 293T cell culture, (B) transfection of 293T cells with pSLCAR-CD19, transfer, envelope, and packaging vectors for lentivirus production, (C) collection of lentiviral supernatant and confirmation of transfection by fluorescence microscopy, (D) infection of Jurkat cells with lentivirus and expansion to establish CAR-J cells, (E) co-culture of CAR-J cells with CD19^+^ B-ALL target cells at an appropriate effector: target ratio for activation assay, and (F) analysis of CD69 expression in co-cultured cells by staining with antibodies (e.g. anti-CD69) and flow cytometry.

Patient-derived mononuclear cells were cryopreserved and stored under standardized biosafety handling conditions in liquid nitrogen until use. Jurkat (ATCC) and 293T (ATCC) cells were cultured in Roswell Park Memorial Institute (RPMI 1640 medium, Cat# SH30027; HyClone Laboratory Tools, Marlborough, MA, USA) and Dulbecco’s Modified Eagle Medium (DMEM; Cat# 11965092), respectively. Both media were supplemented with 10% fetal bovine serum (FBS; Cat# 10500064), 1% penicillin–streptomycin (Cat# 15240062), and 2 mM L-glutamine (from 200 mM stock; Cat# 25030081). All cultures were maintained at 37 °C in a humidified incubator with 5% CO_2_ Fig.1.

### Plasmids

Three CAR plasmids were obtained from Addgene, each based on the validated modular pSLCAR system. All constructs utilize the same anti-CD19 single-chain variable fragment (scFv) derived from the monoclonal antibody FMC63 but differ in their intracellular signaling domains. Specifically, plasmid #135991 encodes the FMC63-28-3z signaling backbone, plasmid #135992 encodes the 41BB-3z backbone, and plasmid #135993 encodes the 3z-only backbone. Each plasmid was co-transfected with psPAX2 (Addgene #12260; second-generation lentiviral packaging plasmid) and pMD2.G (Addgene #12259; VSV-G envelope expressing plasmid) to generate lentiviral particles for Jurkat cell transduction.

### Lentiviral packaging:

On Day 1, 5 × 10^6^ 293T cells were cultured per 10 cm dish (or multiplied by three for a 15 cm dish) in complete DMEM (high glucose, supplemented with 10% FBS and 1% L-glutamine) (Cat# 25030-081 Gibco).

On Day two, plasmid transfection was performed when the 293T cells reached 80–90% confluency. X-tremeGENE™ HP DNA (Cat# 08724121001 Merck) was pre-warmed in a 37 °C water bath and mixed by pipetting up and down. The X-tremeGENE™ HP DNA mix was prepared according to the number of dishes used. A DNA:reagent ratio of 1:3 (μg:μL) was used for all transfections. For each 10-cm dish, 18 μg of plasmid DNA (4 μg pMD2.G, 6μg psPAX2, and 8 μg CAR construct plasmid) was mixed with 54 μL of X-tremeGENE™ HP in 200 μL of serum-free DMEM, incubated for 15 minutes at room temperature,

During the incubation period, the medium in the 293T plates was replaced with pre-warmed (in a 37°C water bath) complete DMEM without penicillin/streptomycin. The old medium was removed by vacuum aspiration. Subsequently, 5mL of full DMEM without antibiotics was gently added to each 10 cm dish, and 15 mL was added to each 15 cm dish by pipetting along the side of the dish to avoid disturbing the cells. Following this, the plasmid mixture was added dropwise to the plates in a circular, clockwise motion.

**Fig.2:**
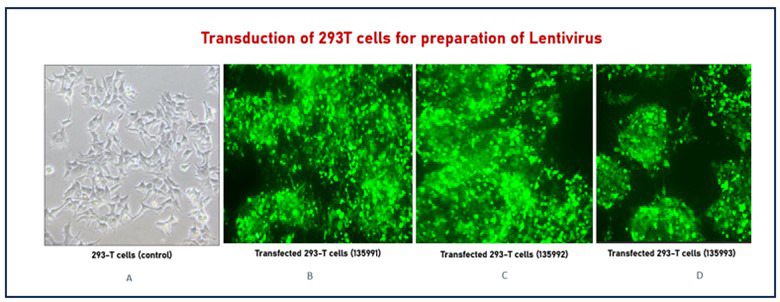
Transduction of 293T cells for lentivirus preparation. (A) Untransfected 293T cells (control). (B–D) 293T cells successfully transfected with plasmids 135991, 135992, and 135993, respectively, showing green fluorescence under a fluorescence microscope.

After 14–16 hours, the medium was replaced with DMEM containing 5% FBS, 1% L-glutamine, and 1% penicillin–streptomycin. Transfection was confirmed through expressed green fluorescence protein GFP and green fluorescence under a fluorescence microscope (Fig.2). Viral supernatant was collected after 24 h, followed by a second collection after an additional 24 hour. The harvested supernatants were filtered through 0.45 μm SFCA sterile filters (Corning®, Cat# 431220) and aliquoted for storage at −80°C. All virus preparations were performed in a single batch and aliquoted before freezing.

All lentiviral work was performed in a biosafety level-2 (BSL-2) laboratory under a Class-II biological safety cabinet. Operators wore laboratory coats, gloves, throughout all procedures. Waste materials, including tips, plates, and media, were decontaminated using 70% freshly prepared ethanol and 10% bleach used.

### Lentivirus Concentration:

Filtered viral supernatant was transferred to a sterile container and mixed with Lenti-X Concentrator (Cat#631232) at a ratio of 1:3 (Lenti-X: supernatant) by gentle inversion. The mixture was incubated at 4°C for 30 minutes to overnight, depending on the volume. After incubation, the samples were centrifuged at 1,500 × g for 45 minutes at 4°C. The supernatant was carefully removed without disturbing the pellet. Residual liquid was removed by brief centrifugation or pipetting. The pellet was resuspended in 1/10 to 1/100 of the original volume using complete DMEM. Concentrated virus was stored at −70 °C in single-use aliquots. One small aliquot from each virus was used for titration. All subsequent infections were performed with an MOI=10.

### Infection of Jurkat Cells with Lentivirus:

Jurkat cells were infected with lentiviral particles using polybrene transfection reagent (lot 3997685 Merck USA) to enhance gene transfer efficiency. Cells were counted using Trypan Blue (Cat# 93595) using the manufacturer’s protocol. The volume of cells was calculated based on cell concentration, and the required medium volume was adjusted to reach a total of 0.2 x 10^6^ cells. For each lentivirus an MOI=10 was used. The final volume was adjusted to 1-mL per well. Polybrene was added to achieve a final concentration of 10 μg/mL (2.5 μL from a 4-μg/μL stock). Plates were gently shaken to mix, wrapped in plastic, balanced, and centrifuged at 2,800 × g for 90 minutes at 32°C. Put the plates in the incubator for more than two hours. For supernatant infection, change the medium after two hours. For concentrated virus, add 2mL medium after three hours. The medium was changed the next day by carefully aspirating the supernatant.

For extended culture, infected cells were transferred to larger plates as needed: to a 10 cm dish with 12 mL of complete medium if more than 1 × 10^6^ cells were infected, or to a 15 cm dish with 30 mL of complete medium when higher cell numbers required weekend incubation.

**Fig.3:**
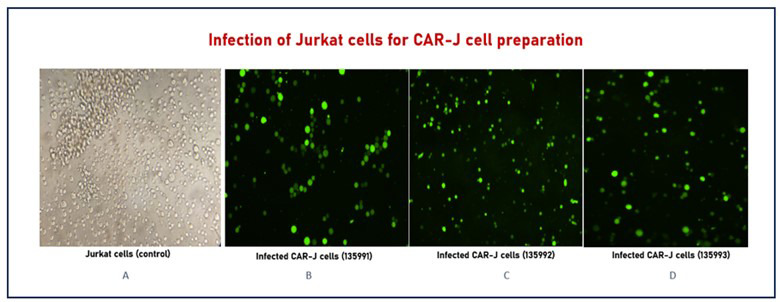
Infection of the Jurkat cell line for CAR-J cell preparation. (A) Control Jurkat cells. (B–D) Jurkat cells successfully infected with lentiviral constructs 135991, 135992, and 135993, respectively, showing green fluorescence under a fluorescence microscope.

Successful infection of Jurkat cells was confirmed by green fluorescence under a fluorescence microscope, as shown in Fig.3.

### Co-culture and Activation Assay:

Infected Jurkat cells (CAR-J cells) were co-cultured with CD19-expressing B-ALL cells isolated from confirmed leukemia patients, as verified by flow cytometry. Jurkat cell activation was evaluated by fluorescent antibody staining and flow cytometry, detecting GFP (FITC) and surface CD69 expression using an APC-conjugated anti-human CD69 antibody (BD Biosciences, Cat# 555533).

**Fig-4:**
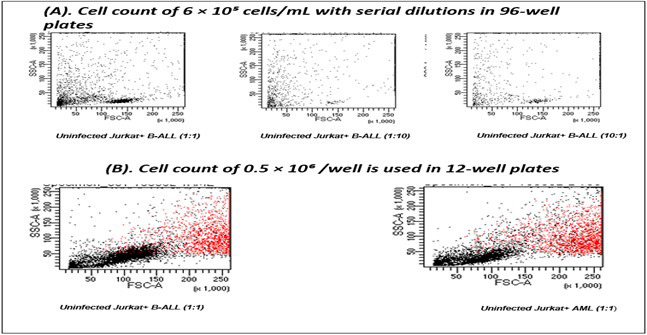
(A) Low cell numbers were detected in flow cytometry when 6 × 10^5^ cells/mL with serial dilutions were used. (B) Increasing the cell count to 0.5 × 10^6^ per well improved cell detection in flow cytometry.

Target cells (B-ALL, AML) were adjusted to 6 × 10^5^ cells/mL and serially diluted (1:10, and 1:100). 50 μL of each dilution was plated in a well of a 96-well round-bottom plate, followed by 50 μL CAR-J cells (6 × 10^5^ cells/mL) in each well to achieve E: T ratios of 1:1, 10:1, and 100:1.The initial results were not satisfactory as cell count was too low for clear interpretation, as shown in Fig.4(A).

To address this, we increased the cell count to 0.5 × 10^6^ (E:T ratio 1:1), and used 12-well plates for co-culture, which yielded better results with a sufficient number of cells to proceed. An E: T ratio of 1:1 was selected as it showed the most optimal results (Fig.4B). Three independent patient-derived B-ALL samples were run in triplicate.

### Flow cytometry methodology & reporting with CD69 Detection:

Flow cytometric analysis was performed using an 8-color, 10-parameter *BD FACS Canto II* flow cytometer equipped with three lasers (blue 488 nm, red 633 nm, and violet 405 nm) with a 4+2+2 fluorochrome configuration. Instrument optimization and fluorescence compensation (spectral overlap), especially for FITC and APC channels was performed using BD comp beads. BD C,S &T beads were analyzed prior to experiment run to ensure optical alignment, Fluorochrome standards & laser performance were within limits. FITC and APC were channels used for event detection, with PMT voltages set to 438 V (FITC) and 475 V (APC), respectively.

Live Jurkat cells were stained with APC-conjugated anti-CD69 antibody at 37 °C for 30 minutes. Cells were then fixed with 1% formaldehyde and immediately analyzed by flow cytometry, following the procedure described by Bloemberg et al., 2020. The gating strategy followed the sequence: FSC/SSC → singlets → GFP^+^ CAR-J cells → CD69^+^ cells. Debris was excluded in FSC/SSC gate. A minimum of 10,000 events were acquired per sample using FACS Diva software. Data were analyzed using FACS Diva, and results reported as percentage mean (geometric) fluorescence intensity (MFI) in the APC channel.

### Statistical analysis:

Statistical analyses were performed using GraphPad Prism version [X]. Data represent the mean ± standard deviation (SD) from three independent patient-derived B-ALL samples, each analyzed in triplicate (technical replicates). Each experiment was repeated at least twice to ensure reproducibility. Comparisons were made using t-test. Statistical significance was defined as: P<0.05 (*), P<0.001 (**), P<0.0001 (***), and P<0.00001 (****).

## RESULTS

### CD69 upregulation in CAR-J cells:

Three sets of experiments were performed, in which all three infected CAR-J cells were cocultured with B-ALL and tested in triplicate, as shown in Fig.1-2.

**Fig.5:**
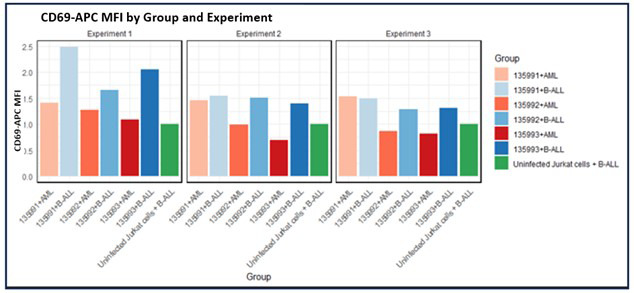
Three experiments were conducted to assess CD69 expression levels for three infected CAR-J compared to controls. In the bar plots, the color coding represents the groups as follows: light pink for infected CAR-J 135991 + AML, light blue for plasmid 135991 + B-ALL, light brown for plasmid 135992 + AML, blue for plasmid 135992 + B-ALL, brown for plasmid 135993 + AML, dark blue for plasmid 135993 + B-ALL, and green for uninfected Jurkat cells + B-ALL.

In the first experiment, the fold change in mean CD69 expression were 2.5 and 1.8 for CAR-J 135991, 2.0 and 1.2 for 135992, and 2.0 and 1.9 for 135993, respectively, compared with controls (uninfected Jurkat + B-ALL, Infected Jurkat + AML, respectively) as shown in Fig.5.

In the second experiment, the fold change in mean CD69 expression was1.6 and 0 for 135991, 1.5 and 1.5 for 135992, and 1.4 and 1.9 for 135993 as compared with the controls. (uninfected Jurkat + B-ALL, Infected Jurkat + AML respectively)

In the third experiment, the fold change in mean CD69 expression was 1.5 and 0 for CAR-J cell 135991, 1.3 and 1.5 for 135992, and 1.3 and 1.6 for 135993 as compared with the controls (uninfected Jurkat + B-ALL, Infected Jurkat + AML, respectively).

**Fig.6:**
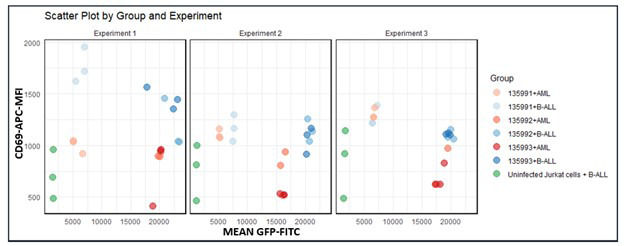
Three experiments were performed to compare CD69 expression (Mean APC) and FITC levels across three plasmids and controls. Color coding represents groups: 135991 (light pink = AML, light blue = B-ALL), 135992 (light brown = AML, blue = B-ALL), 135993 (brown = AML, dark blue = B-ALL), and green for uninfected Jurkat + B-ALL.

Across all experiments, mean FITC levels for Infected CAR-J cells 135991, 135992, and 135993 were 5, 14, and 14, respectively—consistently higher than controls, with 135992 and 135993 showing the greatest increases (Fig.6).

**Fig.7:**
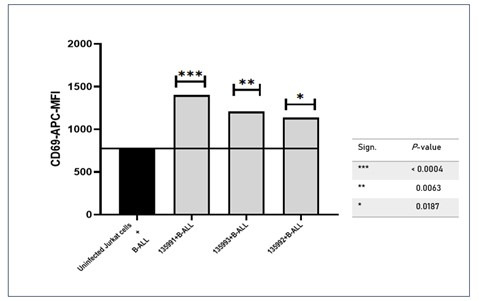
CD69 expression levels in infected CAR-J cells co-cultured with B-ALL cells (135991, 135992, 135993) are compared to both AML controls and the uninfected Jurkat + B-ALL control. All three constructs showed increased mean CD69 APC levels, with statistically significant differences: 135991 (***p < 0.0004), 135992 (**p < 0.0063), and 135993 (*p < 0.0187). *** represents high significance. (p < 0.0001), ** represents moderate significance. (p < 0.001) * represents Low significance (p < 0.05), ns represents no significance (p > 0.05)

The mean CD69-APC MFI of all three CAR-J cells (135991, 135992, and 135993) showed increased CD69 expression compared to the uninfected Jurkat + B-ALL control. The differences were statistically significant, with p-values of ***p < 0.0004 for 135991, **p < 0.0063 for 135992, and *p < 0.0187 for 135993, as shown in Fig.7.

**Fig.8:**
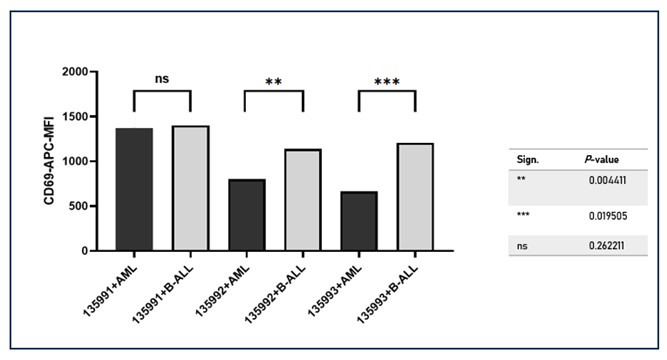
In this figure, each plasmid group (135991+B-ALL, 135992+B-ALL, and 135993+B-ALL) is compared with its respective control (135991+AML, 135992+AML, and 135993+AML). *** represents high significance.(p < 0.001), ** represents moderate significance(p < 0.01), ns represents no significance (p > 0.05).

These results indicate that the binding of all three Infected CAR-J cells to patient B-ALL cells leads to an increase in CD69 expression. However, the level of increase is lower than that observed in positive cell line cultures documented in different studies. Among the three Infected CAR-J cells, 135992 and 135993 exhibit higher CD69 expression compared to 135991. Fig.8 compares the mean CD69-APC MFI values of B-ALL co-cultures with three Infected CAR-J cells (135991, 135992, and 135993) to their respective AML controls. For Infected CAR-J cell 135991 coculture with BALL, the difference from Infected CAR-J cell 135991 coculture with AML (control) was not statistically significant (ns, p = 0.0002). In contrast, 135992 and 135993 showed highly significant differences between B-ALL and AML co-cultures (p < 0.0001 for both). These findings indicate that 135992 and 135993 induce significantly higher CD69 expression in B-ALL cells compared to AML cells, suggesting a plasmid-specific enhancement of CD69 expression in the B-ALL context.

## DISCUSSION

Due to the high response rates observed in leukemia patients treated with anti-CD19 CAR-T cell therapy, numerous clinical trials evaluating anti-CD19 CAR-T cells are currently ongoing worldwide.^12^ As a result, a wide range of anti-CD19 CAR constructs have been developed, many of which remain under preclinical or clinical evaluation. Efficient and reproducible screening strategies are therefore required to prioritize CAR candidates before resource-intensive testing in primary T cells. The CAR-J approach for screening CAR constructs has been previously described. Darowski et al. established an NFAT-based anti-P329G CAR-J reporter system to assess CAR activation.^13^ Jost et al. employed a TCR-like CAR-J approach for antibody detection,^14^ while Jahan et al. developed FiCARs using Jurkat-based platforms.^15^ While several established protocols employ primary T cells for functional screening of CAR constructs, the CAR-J platform offers a rapid, reproducible, and cost-effective alternative for the initial comparative assessment of CAR signaling and activation. Consistent with these studies, we applied a CAR-J methodology in our study to evaluate CAR activation and binding efficacy.

In this study, we screened three anti-CD19 CAR molecules using a CAR-J methodology. However, unlike most published CAR-J studies, which used immortalized antigen-positive target cell lines such as NK-92, Nalm-6, or Raji, we used primary, patient derived mononuclear cells as targets. Bloemberg et al. co-cultured CAR-J cells with CD19-positive cell lines and assessed activation via CD69 expression.^12^ Similarly, Kang et al. identified potent CD19 scFv using an NK/T-cell–based screening platform with established cell lines.^16^ The approach of utilizing patient-derived B-ALL cells as target cells may provide a more clinically relevant evaluation of CAR activation. In our study, two CAR plasmids demonstrated measurable activation; their CD69 expression levels were lower than those reported in studies using immortalized target cell lines. This difference is likely attributable to the use of standardized, highly antigen-expressing cell lines rather than heterogeneous primary patient samples. These discrepancies may also be explained by other factors, including the biological complexity of patient-derived leukemia cells, variability in antigen density, and differences in the tumor microenvironment.

Although the present study focused on CAR activation assessed through CD69 expression, additional functional assays are commonly employed to comprehensively evaluate CAR performance and will be important for future validation of selected CAR candidates. Cytotoxicity assays using luciferase-expressing CD19^+^ target cells, such as NALM-6, are widely used to quantify CAR-mediated target cell killing by primary CAR-T or CAR-NK effector cells in a dose- and time-dependent manner. These assays provide a direct functional readout of antitumor efficacy that cannot be assessed using Jurkat effector cells, which lack cytolytic capacity.

Cytokines such as interleukin-2 (IL-2) and interferon-γ (IFN-γ) are routinely applied to evaluate CAR-induced immune activation following antigen engagement. Such assays enable assessment of CAR activation and provide complementary information to surface activation markers, including CD69. Arvå et al. state that both types of markers are commonly used in combination to comprehensively evaluate T-cell activation and function in response to antigen stimulation.^17^ In this initial optimization phase, we focused on CD69 as an early activation marker. Cytokine assays, however, would be an important addition to the experiment design, to be considered for any future work.

Similarly, at the molecular level, Alter AI et al, state that western blot analysis of CAR signaling components, such as CD3ζ phosphorylation, is frequently used to confirm intracellular signal transduction and to compare signaling strength across different CAR designs. Furthermore, in vivo xenograft models employing immunodeficient mice engrafted with luciferase-expressing leukemia cells represent the gold standard for evaluating antitumor efficacy, persistence, and safety of CAR-T cells in a physiologically relevant setting.^18^

While the above assays were beyond the scope of the current CAR-J–based screening study, their integration with the present findings will be essential in future work to validate and prioritize CAR constructs for translational and clinical development. Further optimization and investigation will therefore be required to enhance CAR-J assay performance and to better understand the factors influencing CAR activation in clinically relevant models.

### Limitations:

Control experiments using standard cell lines (such as Nalm-6 or Raji) were not included for direct comparison. This omission limits our ability to evaluate the true in vitro differences in CD69 expression between patient-derived B-ALL cells and established cell lines under identical experimental conditions, as discussed earlier.

Each experiment was performed using freshly isolated B-ALL cells from different patients. Biological heterogeneity among patient samples, including variations in antigen density and immune cell status, may have contributed to differences in CAR-J activation and CD69 expression levels in different replicates. We did not perform CD19 blocking or knockout experiments in this study due to use of patient material instead of cell lines, which are very challenging to infect. Additionally, limitations in the available funding precluded additional testing through knockout experiments or CD19 blocking agents.

## CONCLUSION

In this study, evaluated a method which may have potential application in the screening of a broader range of CAR molecules for efficacy. Two anti-CD19 CAR-J cells used in our study showed a detectable increase in CD69 expression compared to control (infected Jurkat with AML), while one showed no activation. All three anti-CD19 CAR-J cells show an increase in CD69 expression compared to control uninfected Jurkat cells cocultured with B-ALL.

### Recommendations:

Furthermore, only CD69 expression was measured as an activation marker, without functional cytotoxicity or cytokine assays. The findings are based on in vitro experiments and may not completely reflect in vivo behavior of CAR-J cells. Future studies involving animal models or clinical trials will be necessary to validate these observations in a physiological context

### Authors’ Contribution:

**SH:** Manuscript drafting, experiments, acquisition of data & is responsible for the integrity of the research.

**MZ:**:Did the experiment and acquisition of data

**JA:** Conceived, designed, and edited the manuscript.

**TI:** Acquisition of data, analysis, and manuscript review

**YR:** Critical review and approved the manuscript.
